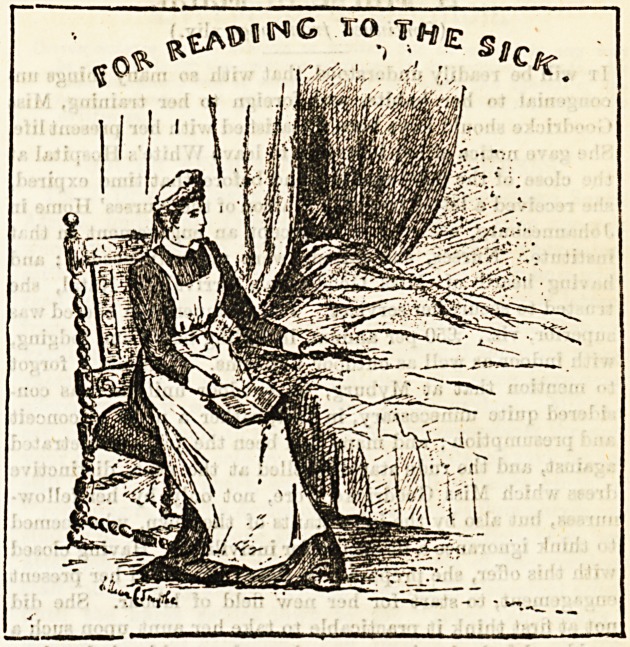# The Hospital Nursing Supplement

**Published:** 1891-11-28

**Authors:** 


### "The Hospital" Nursing Mirror.

Being the Extra Nursing Supplement of "The Hospital" Newspaper.

Contributions for this Supplement should be addressed to the Editor, The Hospital, 140, Strand, London, W.C., and should have the word "Nursing" plainly written in left-hand top corner of the envelope.

### En Passant.

Patients or Friends?—A very sad case has just come to our notice, in which a nurse wrote to a home for nurses and asked if she might bring a friend down. In the course of a few days "the friend" threw herself out of the window, was removed to a hospital, and died. Verdict, suicide during an attack of temporary insanity. The nurse acknowledged that a former attempt at suicide had been made, that the friend was really her patient, and was suffering from hysteria and religious mania. Now, this was a case of deceit; it is also a case on which legal steps might have been taken, and we are astonished that any nurse could have lent herself to a course which was sure to injure the home which had so kindly opened its doors to her. Of course, the other inmates of the home received a shock which quite ruined their holidays. We have not heard whether the head of the home intends taking legal proceedings against the nurse, but at least this should prove a serious warning both to nurses, and to managers of homes.

Kettering News.—About one hundred persons lately attended a meeting at Kettering, held with the object of extending the lately-formed District Nursing Society, Dr. Dryland in the chair. The report of the Committee was presented, showing that from July 28th to October 28th there had been thirty cases on the books, the whole of them requiring great care and skill in the nursing, and many of them taking up as much as four and even five hours a day of the nurses' time. Amongst the cases that had been nursed were typhoid, pneumonia, bronchitis, dropsy, consumption, burns, scalds, abscesses, some requiring two, some three visits from the nurse daily. Miss Wright, the Nurse-Superintendent, was highly complimented. Mr. J. T. Iliffe then moved, "That, in the opinion of this meeting, an hospital is urgently needed in Kettering, and it is desirable at once to start a fund for the purpose of erecting one." This was seconded by Mr. Simons, and supported by many of those present, finally being put to the meeting and heartily carried. A committee was formed to take preliminary steps, and doubtless Kettering will soon have its cottage hospital.

Cane Hill Asylum.—By the kind permission of Dr. Moody, Superintendent of the Cane Hill Lunatic Asylum, and with his personal escort, one of our staff was recently conducted over this splendid building, which contains 1,100 patients. Another wing is now being added; it will be opened in April, 1892, and is capable of holding 900 more. Notwithstanding the vast numbers of the unfortunate inmates, all one's old-fashioned preconceived notions of a dwelling for the insane is, on entering, immediately banished from the mind. Instead of dreary cells, the wards are large and airy, furnished with every comfort, and made bright and attractive by huge masses of flowers, the patients walking about freely, and amusing themselves with games of various kinds, musical instruments, and pet birds and animals. We had a creeping shudder, and a slight feeling of dismay, while walking round a large room at being told there were 140 of the very worst cases in the apartment. The attendants, however, kept very close to us—we need hardly add we kept very close to them—while the doctor, a host in himself, would have speedily brought back order had any outbreak taken place. Nothing unpleasant occurred, however, and we left the building with admiration for the excellent management displayed.

Wolverhampton.—The Matron of the Wolverhampton Eye Infirmary has resigned her post under trying circumstances. Charges were brought against her which she was never given an opportunity of denying, and so she has taken the dignified course of retiring, but holds that the Committee are to blame. The house surgeon has also resigned, so it is time the subscribers demanded some account of their actions from the Committee.

British Nurses' Soiree.—The annual conversazione of the Royal British Nurses' Association will be held on Friday next at half-past eight p.m., at Prince's Hall, Piccadilly. Princess Christian is expected to preside, and present badges to the members. A large and enjoyable gathering is expected, and it is with pleasure we are able to give our hearty approval to this item in the programme of an Association, whose other objects we are obliged at times to criticise.

Doctors and Nurses.—Why may not doctors show their appreciation of a nurse whose skill has been demonstrated to them without being accused of jobbery? Says a provincial paper: "That there is a great deal of jobbing between doctors and nurses I have frequently heard, but that is one of the dark sides of the medical profession, like its trades unionism and many other unworthy things which I need not mention here." This remark is called forth by the wail of a monthly nurse out of work, who goes so far as to mention "bribery" as one of the causes why certain nurses are always sent for by doctors. All this is certainly rubbish so far as decent doctors are concerned or nurses of any worth. We hold that a doctor does well to support those nurses he personally knows to be capable.

Short Items.—Some of the Sisters just sailing for India are to be stationed at Allahabad, where there is a good deal of sickness, but where they have never before had lady nurses.—The Victoria Hospital at Hull is now in excellent condition, the Matron having taken great trouble to secure order everywhere, especially in the kitchens.—Nurse Davis has been sentenced to 20 years' penal servitude for performing an illegal operation.—The Girls' Friendly Society has opened at 21A, Sloane Gardens House, a branch for Northern and Central Europe. Nurses going on to the Continent might find it useful to first seek information from the G.F.S.—Miss Ward, known as Sister Edith, and Miss Hallam, known as Sister Victoria, are nursing Prince George of Wales. Both hold certificates of the Royal National Pension Fund, presented to them by the Princess of Wales.—Miss Alice Richardson was married on November 18th, from the Southend Victoria Hospital, of which she was Matron.—Miss Belchier requests us to state that she at present engaged in lecturing in Surrey.—Nurses may look forward to a busy winter; the influenza is already raging in several provincial towns.—A Bible class for hospital nurses is held every Tuesday at the Morley Hall, in Regent Street.

### Nursing Medals and Certificates.

At the Birmingham City Asylum on November 6th, the following attendants and nurses were presented with the first certificates granted by the Medico-Psychological Association for Proficiency in Mental Nursing: Mr. G. Lees, head attendant; Attendants Connor, Parkes, Butterworth, and Evans; Nurses Deolin, Parkes, Bearpark, Phillips, Coaling, Mabel Moore, and Mary J. Moore. They were at the same time each presented with a silver medal, to be worn when on duty, given by the Birmingham Asylums Committee. The certificates were presented by Mr. Hardman, Chairman of the Sub-Committee of the Waison Green Asylum, and the medals by Mr. Lloyd, Chairman of the General Asylums Committee. Each of these gentlemen addressed a few appropriate words of congratulation to the successful candidates. The medal was designed by Dr. Watson, senior Assistant Medical Officer.

### The Princess of Wales and the Nurses.

The nurses of the R.N.P.F. will be pleased to hear that the screen to be presented by them to the Princess of Wales is now completed, and will be placed amongst the birthday gifts to H.R.H., at Marlborough House on December 1st. Nurses will quite understand that owing to the illness to Prince George, a formal presentation cannot be made. Mr. Wainwright, Treasurer of St. Thomas's Hospital, has kindly permitted the use of the grand committee room at the hospital, where Miss Pritchard will be glad to show the screen to nurse subscribers between 11 a.m., and 4 p.m. on Monday, November 30th. Subscriptions from the following nurses are acknowledged, which were too late for previous publication: E. A. Wildman, 10s.; Rosalind Paget, 2s.; E. A. Abel, 2s.

### Nursing in Denmark.

Amongst the many papers of interest circulated by the late Congress of Hygiene and Demography was a bulky volume on the medical organization of the country of Denmark. It included a very instructive chapter on nursing, part of which was from the pen of Mrs. Gordon Norrie, who has before now written in English papers on the subject. It seems that the private nurses in Denmark charge by the day at a rate of from 4s. to 6s.; they train in the large hospitals, but the training is merely theoretical, and they have none of the lectures and classes which are our privilege. The largest hospital is the Common Hospital, which is divided into the male and female sides; a separate Matron rules over each side, and under her are two staff nurses and probationers, there being about one probationer to 15 patients; and as there are 70 beds on each side, each staff nurse has to look after 35 patients. There are also 22 housemaids in the hospital. The probationers are not paid for the first few months; the staff nurses receive about £25 a year, the Matrons about £50. The nurses are well housed away from the wards, and have pleasant dining rooms; they are off duty for two hours eaery other day, with a half-day once a week, and a fortnight or three weeks' holiday in the year. Most of the hospitals grant pensions to the nurses who serve them long and faithfully.

The district nursing seems to be mainly supported by a Government subsidy, and to be carried on by women of the people who have had some experience in hospital. There are 87 of these district nursing societies, and they employ 126; several of them charge a small fee for the nurse's services.

Two special institutions, the Red Cross and the Deaconesses' Institution, deserve special mention. The Red Cross was founded in 1875 with the object of supplying trained nurses in time of war, to fulfil which they train nurses in time of peace, and have so far trained 100 women, of whom 60 are still in their service. The time of training is one year, and every winter a course of ambulance lectures are given to the nurses. When trained the nurses are employed by the Society to nurse all classes, and the Society charges for the nurses' services according to the position of the patient, the very poor being cared for gratis.

The Deaconesses' Institution is on the same lines as the well-known Institution at Kaiserwerth, and was founded in 1863 by Princess Louise of Denmark. There are 167 Sisters, and they have homes for invalids, for servants, for children, &c., besides their hospital, which is celebrated for the number of successful gynæcological operations there performed. There is a peculiar system of night nursing, the night being divided into two watches of four hours, and being shared by two sets of nurses.

With regard to midwifery, Denmark is far advanced. Since 1810 the whole country has been divided into districts, and a midwife appointed to each district at a fixed salary. The midwife conducts every case of normal labour, and is not allowed leave any pregnant woman after labour has set in; she is in some manner an officer of state, and has to fill in a printed schedule giving particulars of every case she attends, and forward it to the Board of Health. Midwives must be between eighteen and thirty at the time of their training; they have to serve nine months in a lying-in hospital, where the instruction is theoretical as well as practical. Denmark has several good text-books on midwifery, and also a journal for midwives; whereas the chief nursing text-book is a translation of Miss Nightingale's "Notes."

### Notes from Australia.

(*By Our Own Correspondent.*)

Melbourne, October 13th.

Sister Read, who trained at St. Thomas's, maintains at Sydney a very comfortable Invalid and Nurses' Home. It is a large house on the top of a hill in a fashionable neighbourhood; there is telephonic connection with all parts of the city. Nurses are sent out, and take their own earnings; a staff is also kept to attend to the invalids in the home. Patients from home travelling for their health are received.

There is only one topic here just now—influenza. We've all got it, the papers are full of it, and all work is delayed by it. The nurses of the Melbourne and Alfred Hospitals are particular sufferers, and they have fashionable fellow victims in the Governor and Countess of Hopetown. At Castlemaine are a very large number of patients in the institution suffering from the malady; nearly the whole staff have fallen victims to it. Dr. Colin Henderson, the resident surgeon, fought sturdily against the disease for some time, but at last he was compelled to relinquish his duties and take to his bed, where he still remains, but is recovering from the illness. The cook, day wardsman, night wardsman, night female nurse, the acting day wardsman, and the acting night wardsman, are now laid up with influenza. At the late meeting of the Board of Health there was a discussion about this disease when Dr. Greswell said that many deaths from chest affections were directly attributable to influenza. It was desirable that the fact of the disease being infectious should be more generally known. There was a report by Dr. Parsons on influenza throughout England, and Dr. Buchanan commenting upon it had pointed out that there never was more thorough evidence afforded than as to the disease being infectious in its epidemic form, and communicable in the ordinary intercourse of individuals. He felt that sufficient emphasis had not been given to the necessity for isolation and disinfection in such cases. So far as possible both should be carried out. All handkerchiefs and other linen should be passed immediately through boiling water, while all persons except those attending patients should be kept out of rooms occupied by sufferers from the complaint. The Chairman said that the Board could do nothing further than had been already done by circular. The three broad measures were isolation, disinfection, and lying up.

The death of Dr. Singleton from cystitis removes a greatly respected colonist. He was a Dublin man, but took his M.D. at Glasgow; he was noted for his success during the Dublin cholera outbreak, and in 1850 he came out to Melbourne. He was an active worker in the temperance cause, and a frequent visitor in the gaols; he established in the poorest part of Melbourne a medical mission, and was well-known as one of our most practical philanthropists.

General Booth had a most enthusiastic reception here, and drew hundreds to the penitent form. So many of the members of the Army here had never seen the General, and they welcomed him almost as a prophet. The meetings were crowded, the hymns were shouted, and the "Amens!" rent the air. Such is the latest illustration which has been afforded us of religious emotion, and of that peculiar influence, called by some mesmeric, which lays hold of the individual in a crowded and enthusiastic meeting, and carries him along even in spite of himself.

The election of medical officers to the Women's Hospital has been carried out on our abominable system of votes by subscribers, with the result that the three most competent men for attending to the patients are out, and the three most skilful canvassers are in. On the face of it, it is a man of science—a man in earnest, and in love with his profession, and skilled in his work—who should secure a post in a hospital; not the man who can cajole women out of their votes, and drink endless afternoon teas. The *Argus* and other papers are hitting hard at the system; let us hope they will kill it.

### THE BLESSINGS OF SICKNESS.

It seems a very strange answer to prayer that we should have sickness sent us when our petition has been for work; good sound, hard work is what we think we want, for that alone will lead us on to competence and comfort. Instead of that we are attacked by a very bad illness. Now, we must not be angry and find fault with the Almighty for acting in this way. He knows better than we do what is necessary for our real good. If He had given us plenty of occupation, it is very possible we should soon have been so engrossed by our daily work that we should have forgotten the Author and Giver of all good things. At any rate, we are prevented for the present from going astray till we have learnt the lesson of dependence on God's hand. Our loving Father possibly saw we were sliding away from the right path, not with any direct intention of offending Him, or of setting ourselves up in opposition to His will, but simply that we felt confident we could get on very well by ourselves, and were sufficient for our own guidance in all things.

But have we ever thought how many evil things we have been kept from which we could not avoid for ourselves? How many foolish habits we might have fallen into, how many idle thoughts we should have indulged had we not been shielded by this sickness which we deplore? Our loving Father hid us from these temptations; He took us into His own special care. On the sick bed we are safe from the provoking of all men; God keeps us secretly from the strife of tongues. We are being taught real living truths, for we want good food to feed upon, not husks, and we are gathering these advantages from a closer intercourse with God. To see our faults is very painful, but very wholesome. Where, except in the quietness of the still chamber, should we have thought of the selfishness of our past lives, of the little gratitude we have shown for the love and tenderness of our parents and friends. We can see, too, that though we have said a few prayers and gone to a place of worship when it was not inconvenient, yet it was but a form, the true spirits of love to our Saviour and an earnest desire to serve Him were absent from our hearts.

Now by this suffering we are learning, or ought to be learning, to be thankful and willing to suffer if, our motives are by this means purified, and when it shall please our Heavenly Father to restore us once more to health, we shall thank Him nightly for the work He has enabled us to do in the day; for the work He has given, and the strength He has added to perform it.

We may not know from whence our great happiness proceeds, but we are bringing into our daily life the knowledge that no duty is too small or mean to be overlooked, and not one which can be properly set apart from the service of God. With such a store of wisdom thus acquired, those about us will take notice that we have been with Jesus.

### A Nurse in Natal.

(*Continued from page* xliv.)

It will be readily understood that with so many things uncongenial to her habits, and foreign to her training, Miss Goodricke should have been dissatisfied with her present life. She gave notice of her intention to leave White's Hospital at the close of the third month; and before that time expired, she received a letter from the Matron of the Nurses' Home in Johannesburg, asking her to accept an engagement in that institute. Nurses, she stated, were urgently needed; and having heard of Miss Goodricke's arrival in Natal, she trusted to secure her services. The remuneration offered was superior, viz., £50 per annum, including board and lodging, with indoor as well as outdoor uniforms. (I think I forgot to mention that at Myburg, an outdoor uniform was considered quite unnecessary, in fact, rather a mark of conceit and presumption; and many had been the jokes perpetrated against, and the rude stares levelled at the neat, distinctive dress which Miss Goodricke wore, not only by her fellow-nurses, but also by the inhabitants of the town, who seemed to think ignorance a fit excuse for incivility.) Having closed with this offer, she prepared, on the expiration of her present engagement, to start for her new field of labour. She did not at first think it practicable to take her aunt upon such a rapid and fatiguing journey as she understood lay before her.

But Mrs. Thornton would not now hear of remaining behind. She felt that any privation or hardship was preferable to being left alone in a place where her retired habits and their recent loss had prevented her from making new friends.

They therefore started together on their journey to Johannesburg, that wonderful "Golden City" which had sprung up so marvellously in the Transvaal Republic.

And now, indeed, a new world of wonder and interest lay before them! The first part of their journey, from Myburg to Ladysmith, was made by rail, to which succeeded three days' travel by mail coach. Each day's journey occupied about fifteen hours, from five a.m. to eight p.m., the only stoppages during this time being for meals, from a quarter of an hour to twenty minutes.

Their route lay sometimes over the worst conceivable roads, and poor Mrs. Thornton thought she could never survive it, as they tore up hills and rushed down dales, or ventured into swollen, bridgeless rivers, where she expected every moment to be her last. Yet she was bravely determined to go wherever her beloved niece went, and to die with her if need be. Their first day's journey lay over the Drakensberg Mountains, where they had splendid glimpses of a wild bushy country, interspersed with many stony tropics, while behind them lay a lovely panoramic view of fair Natal.

The second day was spent in passing through the Free State—that bleak, barren land of splendid pasturage and viewless tracts. The third day's journey, like the second, was uninteresting until they approached Johannesburg, shortly before sunset. For miles before their arrival, and, indeed, before they came in sight of the city, a wonderful roar fell upon the ear, and became ever louder and louder, like the roaring of the sea upon the shore.

"I did not know that we were coming near to the coast," said Mrs. Thornton to the driver, timidly.

"No more we are, ma'am," he replied; "that's the batteries you hear, not the sea. You won't find this a dead-and-alive place like Myburg; always lots of life going on here. And there's many will be glad enough to see you, Miss," he added; "there's many a poor fellow would give a bag of gold to have one of your sort beside him when he's down."

"Is there much sickness just now?" asked she.

"Any amount, Miss. Doctors and nurses got their hands full. They know how to value a good nurse here, that they do!"

As they came in sight of the city, the sun was setting behind it, beautifully illustrating the name given to it for another cause. As the warm, bright rays flooded every street and building, it looked, indeed, in that transient glory, like a veritable "golden city."

Johannesburg, at that time, seemed to be built without any fixed plan. The long, straggling streets had apparently no uniformity, and buildings of all sizes and descriptions stood side by side—the substantial stone or brick edifice, and the filthy mud hovel in closest proximity. Our travellers could not but be struck by the extraordinary number of "canteens," or taverns, as we call them in this country, one passenger counting no less than forty of these alluring dens while the coach was passing up the main street. It has been computed that there were as many as two hundred of these buildings in the entire city.

At the period of which I write, although the first great "boom" was even then over, there was another just setting in, and everything was in full activity, the population being about 20,000. Having seen her aunt to a room, for which they had previously telegraphed, Miss Goodricke took a cab, and proceeded to the Nurses' Home, where she received a very kind and cordial welcome. This temporary Home consisted of a long, brick building, in which two nurses, for want of space, occupied a room together. Although this was a happy improvement upon her late quarters, it was not—in this more enlightened country—considered a desirable arrangement]; and later on, when a new and more commodious home was erected in Government Square, a separate room was allotted to each nurse.

The original Home for Nurses, of which Miss Goodricke became a member, was controlled by a committee of thirty-four ladies and one clergyman, and was presided over by a Matron. It was managed, to a great extent, like similar institutions in this country. The nurses were guaranteed a home and salary, whether in work or not; and people applying for, and obtaining nurses from the Home, paid for their services at the fixed rate of £4 4s. per week, a sum, be it remarked, not only cheerfully but gratefully paid.

At this time there was no district nurse for poor people in Johannesburg, but as there may be said to have been no poor in the city, then this want was not felt till later on, when district nurses were promptly supplied by one of the church organisations. Miss Goodricke had only enjoyed one night's rest after her journey when her services were called into requisition. Her first case was one of pneumonia, and, as soon as her patient was out of danger, she was asked to take a case which was so typical of the country, it deserves a chapter to itself.

(*To be continued.*)

### A Bed for a Sick Nurse.

Up to November 22nd we had received the following further sum towards the bed for a sick nurse: Mr. A. Rosling, one guinea; K. M. S., 1s. 6d.; Mrs. Bragge, 1s. 6d.; Nurse Attfield, 1s.; one of the first thousand (Leith), 2s.; 533 Fitzroy, 2s.; Two Little Helps, 2s. This gives us the full 30 guineas we wanted, and £1 over in small sums, which we propose to hand over to help furnish the room in a comfortable manner. And now to explain to the kind supporters of this scheme what is being done. The Brassey Home at St. Leonards is a holiday home for nurses—not a luxurious one, but a pleasant, bright place, on homely lines, where the nurses are received for 15s. a week. It is not a charity in any way, and is not supported by either Lord or Lady Brassey as has been stated; both have been, and we hope ever will be, appreciative and helpful friends of the Home which bears their name, but the Home is entirely managed by Miss Holditch and Sister Frost. Two or three cases of nurses needing change, but very poor after fighting through an illness, came to our notice, and twice the managers of the Brassey Home received such cases free of expense. Feeling we could not trespass so much on their generosity, we asked our readers for 30 guineas, which Sister Frost considered would pay for a free bed at the Home. These 30 guineas have now been subscribed, the little room set apart for this scheme is being repapered and decorated, and from January 1st, 1892, nurses will have a free bed at the disposal of any member of their ranks who is too poor to secure rest and sea air for herself. Such nurses will have medical attendance free, but medicine and extra diet will have to be supplied from a special fund. Nurses will also be able to have reduced fares from London. All applications for the use of the bed must be made to Miss Holditch, and must be accompanied by a letter from the Matron, stating that the case is suitable, if the applicant is working in a hospital or a home; or by a doctor's certificate if the applicant is working on her own account. Preference will be given to cases recommended by subscribers, and in case any difficulties should arise a small committee, consisting of Nurse Elms, Miss Holditch, Sister Frost, Miss Pritchard, and the editor of the "Nursing Mirror," has been formed, to whom all debateable points will be referred. We have personally visited and inspected the Brassey Home, and noted the economical and clever management there; also the fact that the Home is healthily situated. We hope not many nurses will need to apply for the bed, but we are sure that those who do will appreciate the benefit of staying in such a bracing place.

### Notes and Queries.

To Correspondents.—1. Questions or answers may be written on post-cards. 2. Advertisements in disguise are inadmissible. 3. In answering a query please quote the number. 4. A private answer can only be sent in urgent cases, and then a stamped addressed envelope must be enclosed. 5. Every communication must be accompanied by the writer's full name and address, not necessarily for publication. 6. Correspondents are requested to help their fellow nurses by answering such queries as they can.

### Queries.

*Conjuring.*—Can anyone give me the address of a good conjuror for our Christmas treat?—A. C. C.

### Answers.

*Alice.*—Mrs. Creighton Hale, 3 guineas (special terms for nurses). The West-end School of Massage 10 guineas. See our advertisements.

*Probationers in Cottage Hospitals.*—It is not usual for the Committee to engage a probationer without submitting the selection to the Matron. The Matron in all good hospitals has the power of engaging and discharging the nurses, subject to the approval of the House-Committee to whom she reports all such occurrences.

*F. E. Ford.*—There is no such hospital, and very rightly. You had better try for the Diploma of the London Obstetrical Society. Classes to prepare for this are held at the Nurses' Club, 12, Buckingham Street, Strand, W.C.

*Sister Dorothy.*—For full particulars get "The Hospital Annual"; we have no room to give them here. If you have "got quite the idea that their nurses are of the lower class," you had better not go to a hospital. The nurses are always a mixed lot, but no class distinctions are admitted amongst them—they are not snobs. Of course you will have to wait to get into a good hospital; the nursing ranks are crowded just now.

*Nurse Britton.*—No; your skeleton was received but it was that of Nurse Brittan that received honourable mention.

*M. Beaman.*—Apply to the manager, Royal National Pension Fund for Nurses, 8, King Street, Cheapside, London, E.C.

*Inquirers.*—Get Cullingworth's "Manual for Monthly Nurses," Lewis's "Text-book of Nursing," and Huxley's "Physiology."

*Christmas Competitions.*—Parcels received from Nurse Fernie, "Jenny Wren," "Scots Janie," Nurse Ritchie, and "Dyke Road, Brighton."

### Everybody's Opinion.

*Correspondence on all subjects is invited, but we cannot in any way be responsible for the opinions expressed by our correspondents. No communications can be entertained if the name and address of the correspondent is not given, or unless one side of the paper only be written on.]*

### SOUTH AFRICA AND ENGLISH NURSES.

Dr. J. Duncan Greenlees writes from Grahamstown, South Africa, under date October 26, 1891: Some time ago you were kind enough to publish a letter of mine in your interesting magazine on the above subject, and to show how wide read you must be, I have received a large number of letters from English nurses, some asking for further information regarding the climate, &c., others enquiring if there are many vacancies in the colonial institutions, and some have even offered me their services as nurses in the asylum. Might I ask your permission to reply to these correspondents through your pages? This colony is young, hospitals and asylums are still few and far apart, and life here still partakes much of "roughing it." The duties of hospital and asylum nurses are similar to those in vogue at home, but in addition, here, less attention seems to be paid to the comforts of the resident staff; accordingly, at first at least, a feeling of discontent arises until the nurse settles down to the "rough and ready" colonial mode of existence. The various hospitals are frequently advertising for experienced nurses, but I am not sure that the authorities aid their passage out if selected in England. Even in Government posts the authorities are very chary in paying passages out, but in the cases of healthy men and women, by means of a Government grant, the shipping companies take out passengers at reduced rates. I have my full complement of attendants and nurses here at present, but should vacancies occur, I will bear in mind several of my correspondents. Male attendants are of no use unless they know some trade, and then there is always a market for their services. The ordinary attendant who devotes his time to promenading the airing courts with his hands in his pockets is not required here; we have plenty of this kind.

In conclusion, permit me to thank my many correspondents for the interest they take in this, the coming colony and for the trouble they have put themselves to in writing me on such a subject. I have to thank you, too, for your magazine, which is extensively circulated out here, and helps to bind the colonial nursing profession with that of home.

### Appointments.

[It is requested that successful candidates will send a copy of their applications and testimonials, with date of election, to The Editor, The Lodge, Porchester Square, W.]

Royal Free Hospital.—After an exciting contest, Miss Henrietta Wedgewood, who has been a Sister at King's College for the last eight years, has been elected Matron of the Royal Free Hospital. Miss Wedgewood trained at the Middlesex Hospital, and has won golden opinions everywhere for her powers of pleasant yet efficient management. She is likely to prove a very popular and worthy successor to Miss Barton.

Southend Victoria Hospital.—Miss Clara Louise Gunn entered on the duties of Matron to this hospital on the 20th inst. She trained at the Hull Royal Infirmary, and has worked at Greenwich and Leith, and as Matron at Coltishall. Miss Gunn's testimonials are excellent, and she is likely to do good work at her new post.

### Wants and Workers.

*Old Linen.*—The Eccles and Patricroft District Nurses (1, Byron Street, Patricroft, near Manchester) are badly in want of old linen for poultices and dressings.

### "Dr. Sutters."

*(Continued from page xlviii.)*

THAT evening he was inwardly convicted of neglect of the social usages. He confessed that he ought to call upon the Vicar after receiving his invitation to dine. It would be something of a trial to gossip for several hours with three vivacious girls who would enjoy quizzing him; but it was a matter of politeness, even duty. So the next day he walked up to the Vicarage about dusk. He hoped to escape to his library and his insects after afternoon tea. The maid said that "Mr. Norton was in London, but Miss Mabel was within," and as there was no possibility of any but an invidious construction being put upon his retreat by that young lady, he allowed the servant to take his card. While he waited in a pretty little room that opened on to a sloping lawn with crocus beds, he saw an open portfolio of water-colour sketches upon a table. The uppermost drawing was a portrait of the landlord of the "Farriers' Arms." It was a clever semi-caricature, and underneath was written, "Our Local Celebrities. No. 1.—Our Boniface." In the corner were the initials M. N.

"So this is Miss Mabel's work," murmured Dr. Sutters, holding the portrait at arm's length. "Very clever, I'm sure. And who is number two? Hulloa! 'Dr. Sutters, our Distinguished Beetle Hunter.' Come, that's too bad! I'll be hanged if my nose approaches those dimensions. And what a parody on my whiskers! Confound it! they're not scarlet! How they giggle over this when people come to to tea. Well, I don't mind fun, but there is malice in this thing. Now I know the meaning of that smile when my lady meets me. What's this on the back? 'Dr. Augustus Sutherst, better known as "Old Sutters," is one of the latest importations to our collection of odd fish. He is a great medicine man (on paper). Since his advent in Hook he has attended one case of measles. It would not, therefore, be just to pronounce an opinion upon his skill in the art of healing. But he is greater at beetles than diagnosis; this is the private judgment of the artist, and it is given for what it is worth. Kindly observe the massive brow.' Ah! very funny, very funny, young lady," muttered the doctor, and he was about to say more when the door opened.

With a nervous glance at the portfolio, Mabel Norton bowed, and then sat down, tingling all over. The caricature lay upon the table, and the nose, with its exaggerated prominence, caught her eye. But she was not lacking in *savoir-faire.*

"I am sorry papa is from home," she said calmly. "It's not often that he is away."

"I hope to see him another day, thank you. It is not of much consequence, and we are all near neighbours. What charming spring weather. I have been admiring your crocuses."

"Papa takes a great interest in gardening."

Then the nose obtruded itself upon her once more, and she flushed pink in the twilight. "What can he think of me?" she asked herself. It was as well that she remained in ignorance upon that point, for at that moment Dr. Sutters thought her a pretty minx, and no more nor less. Much as he longed to avenge himself upon his caricaturist, he was too gentlemanly to speak about the portfolio of celebrities.

"You will have a cup of tea, Dr. Sutt—Dr. Sutherst?" she said.

"Thank you, I'll not trouble you," he answered, enjoying her discomfiture at having almost let slip the garbled rendering of his patronymic. "I will look in next week, when Mr. Norton is at home. I hope you are all well, and don't need the doctor? Unless your healthy colour tells fibs, you have no need of him."

"Can he see my blushes?" she asked. "Oh! how I hate him."

After this the conversational ability of the eldest Miss Norton was doubted by her visitor. She was monosyllabic and ill at ease. Several attempts at polite satire, in return for Dr. Sutters' allusion to her heightened hue, fell flat, and she found herself positively stammering with nervousness. Then came a long, awkward pause, and Dr. Sutters rose, with his hand held out.

"How dare he rummage my pictures!" she said, when the doctor had passed out of the garden. "It was downright impertinence! How stupid of me to leave the wretched things about."

She was sure that the doctor had found amusement in her embarrassment. What a horrid, triumphant smile he gave her when he shook her hand. But never mind. Next time they met she would simply wither him with irony. Here, however, she reckoned without regard to the caprices of fate. A week after, she was walking in Crocombe Lane, looking for violets among the trailing ivy, and humming a waltz, when suddenly a shaggy lurcher's head was thrust through the hedge, and a mouthful of white fangs snapped in her face.

"Go away you disagreeable, disreputable animal," she said, giving the dog a poke with her walking stick.

But the creature was rendered more hostile than before by this method, and, with an alarming growl, he seized her left arm just below the elbow, and bit deep into her flesh. The pain was so acute that it was only by strong control that she was able to resist screaming. Her stick rattled a dozen blows on the brute's head, but she might have beaten iron for all the effect that this had upon him. She was almost fainting with pain and fright, when a pair of spectacles appeared over the hedge, and a rasping voice rated the dog till he dropped the arm. Blood was oozing through her sleeve, and her face was very white, when Dr. Sutters threw down his beetle satchel and scrambled down the bank.

"The vicious brute!" he exclaimed. "It's Manning's dog. I'll have him shot; he's not fit to be at large. Has he hurt you, Miss Norton?"

"I'm afraid I'm badly bitten," she answered, wincing with the pain.

"Let me look." He ripped up the sleeve, and put his spectacles close to the wound. "It's a very nasty bite. You must have it cauterised at once. Take my arm, and come to my surgery. Can you walk?"

"Oh, yes! I'm not so bad as that," she said, trying to smile.

"That's plucky. It's only three minutes' walk."

*(To be continued.)*

### Amusements and Relaxation.

**SPECIAL NOTICE TO CORRESPONDENTS.**

**Fourth Quarterly Word Competition commenced October 3rd, ends December 26th, 1891.**

The word for dissection for this, the NINTH week of the quarter being

"TYPHOID."

| Names. | Nov. 19th. | Totals. | Names. | Nov. 19th. | Totals. |
|---|---|---|---|---|---|
| Lightowlers | 69 | 347 | Jenny Wren | 65 | 263 |
| Bonne | 61 | 3[illegible]9 | Darlington | 63 | 340 |
| Morico | 73 | 400 | Nurse G. P. | — | 99 |
| Robes | — | 143 | Hetty | 63 | 255 |
| Dulcamara | 64 | 354 | Janet | 66 | 299 |
| Psyche | — | 7 | Jackanapes | 70 | 290 |
| Agamemnon | 72 | 38[illegible] | Ex Nurse | — | 99 |
| Nurse J. S. | 65 | 340 | | | |

**Notice to Correspondents.**

JENNY WREN.—No word dissection received that week.

All letters referring to this page which do not arrive at 140, Strand, London, W.C., by the *first post on Thursdays*, and are not addressed PRIZE EDITOR, will in future be disqualified and disregarded.

## Figures and Tables

**Figure f1:**
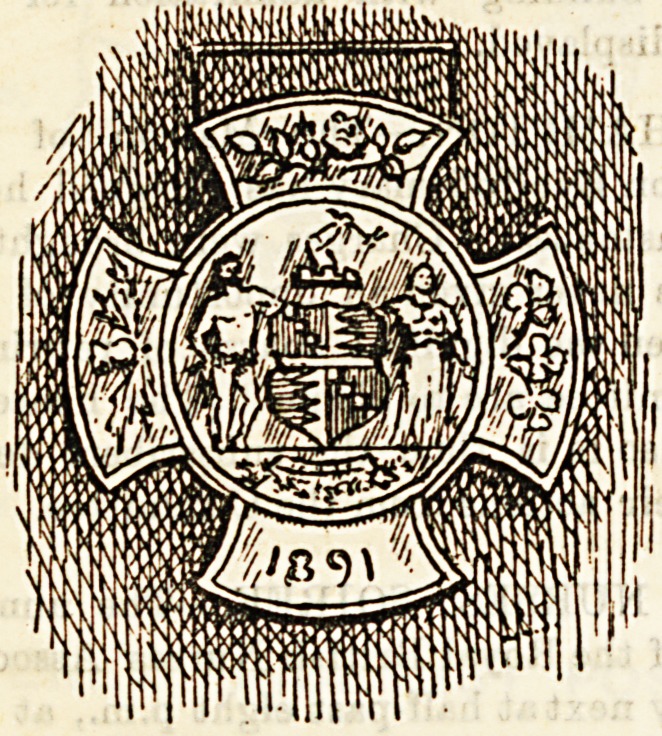


**Figure f2:**
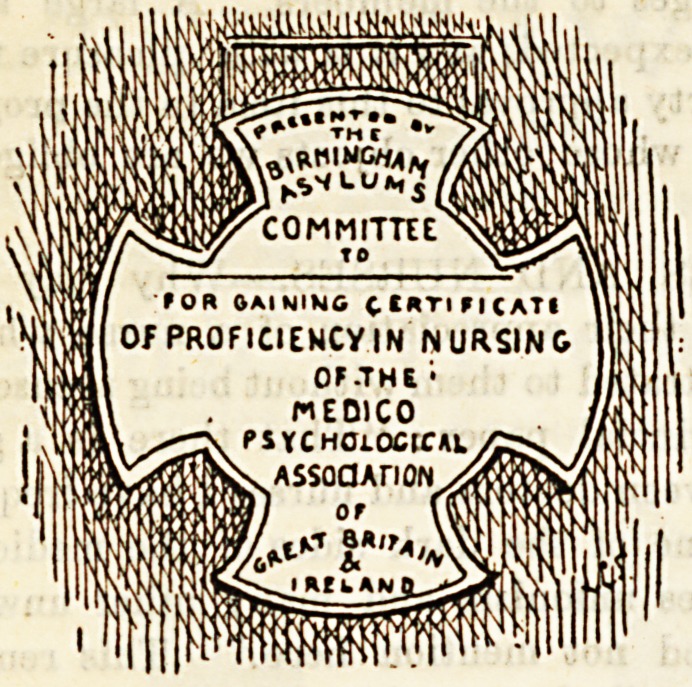


**Figure f3:**